# Pulmonary Syphilis

**Published:** 1917-04

**Authors:** 


**Pulmonary Syphilis.** Cadis Phipps, Boston, Boston M. & S. Jour., Mch. 15, reviews the literature and reports a probable case, showing almost complete recovery on mixed treatment. The clinical picture is that of tuberculosis but rather of mild type.

